# Approach to Secretory Carcinoma of the Minor Salivary Glands of the Buccal Mucosa: A Case Report With a Review of the Literature

**DOI:** 10.7759/cureus.115671

**Published:** 2026-09-02

**Authors:** Zoya Hasan, Meeta Singh, Kirat Kaur, Ravi Meher

**Affiliations:** 1 Department of Pathology, Maulana Azad Medical College, New Delhi, IND; 2 Department of Otorhinolaryngology - Head and Neck Surgery, Maulana Azad Medical College, New Delhi, IND

**Keywords:** buccal mucosa, etv6-ntrk3 fusion, immunohistochemistry, micro- and macrocystic pattern, secretory carcinoma

## Abstract

Secretory carcinoma (SC) is a rare, low-grade malignant neoplasm of the salivary glands, most commonly arising in the parotid gland. Involvement of the minor salivary glands of the buccal mucosa is exceedingly rare, with only a handful of cases reported in the literature. A 30-year-old female presented with a one-year history of a swelling over the left cheek. On clinical examination, a 2 × 2 cm, lobulated, cystic swelling was identified in the left buccal mucosa. Fine-needle aspiration of the swelling showed predominantly eosinophilic material with occasional scattered cells. The lesion was surgically excised and submitted for histopathological examination. Gross examination revealed a gray-brown, soft-tissue mass measuring 2.5 × 2 × 1 cm. On serial sectioning, a well-circumscribed cystic, gray-white lesion measuring 1 cm in diameter was identified. Histopathological examination of the lesion showed features consistent with SC. The tumor cells were positive for GATA3, mammaglobin, S100, and CK7. The postoperative positron emission tomography-computed tomography revealed no tumor deposit, and the patient remained free of local recurrence during the six-month follow-up period.

## Introduction

Secretory carcinoma (SC) of the salivary glands, previously termed mammary analogue secretory carcinoma, was initially identified as a distinct salivary gland neoplasm by Skálová et al. in 2010 [1-3]. Subsequently, the 2017 World Health Organization (WHO) classification of head and neck tumors recognized SC as a separate clinicopathological entity [2]. The tumor is defined by characteristic genetic alterations involving rearrangements of the *ETV6* or *RET* genes, with *ETV6::NTRK3* and *ETV6::RET* fusion transcripts detected in the majority of cases [3].

SC is an uncommon malignancy, accounting for fewer than 0.3% of all salivary gland malignancies [4]. Clinically, it typically manifests as a slowly enlarging, painless mass. The parotid gland is the most frequently involved site, followed by the oral cavity and the submandibular gland. Within the oral cavity, the lip and soft palate represent the predominant locations of occurrence [3].

Involvement of the buccal mucosa is rare, with approximately 32 cases reported in the published literature. Here, we present a rare case of SC of the buccal mucosa in a 30-year-old female.

## Case presentation

A 30-year-old female presented to the Otorhinolaryngology Outpatient Department with complaints of left-sided cheek swelling for one year. The swelling was insidious in onset and gradually progressed to the current size over the course of one year. There was no history of swelling in the neck region or elsewhere. No history of tobacco consumption was reported. Clinical examination of the swelling revealed a 2 cm × 2 cm lobulated, cystic swelling in the left buccal mucosa. It was mobile and firm in consistency. Ultrasonography of the neck revealed no enlarged lymph nodes.

Fine-needle aspiration (FNA) of the swelling yielded yellowish fluid. Smears examined showed predominantly eosinophilic material with occasional scattered cells. The mass was then surgically excised and sent for histopathological examination.

Grossly, a gray-brown, soft-tissue mass measuring 2.5 cm × 2 cm × 1 cm was received. On serial slicing, a cystic gray-white growth was identified measuring 1 cm in diameter. Microscopic examination of the sections showed tissue lined by stratified squamous epithelium, with subepithelial tissue showing the presence of tumor in a macro- and microcystic pattern and lobules, along with foci of stromal invasion (Figures 1a, 1b). The individual tumor cells showed a prominent acinar pattern with a moderate amount of cytoplasm and cytoplasmic clearing with prominent eosinophilic secretions in the lumen (Figures 1c, 1d). No lymphovascular invasion (LVI) or perineural invasion (PNI) was noted. The tumor was reaching up to the deep resected plane; however, the overlying mucosa was uninvolved. Further resection with a 1 cm margin was performed, which showed a free deep resected plane.

**Figure 1:**
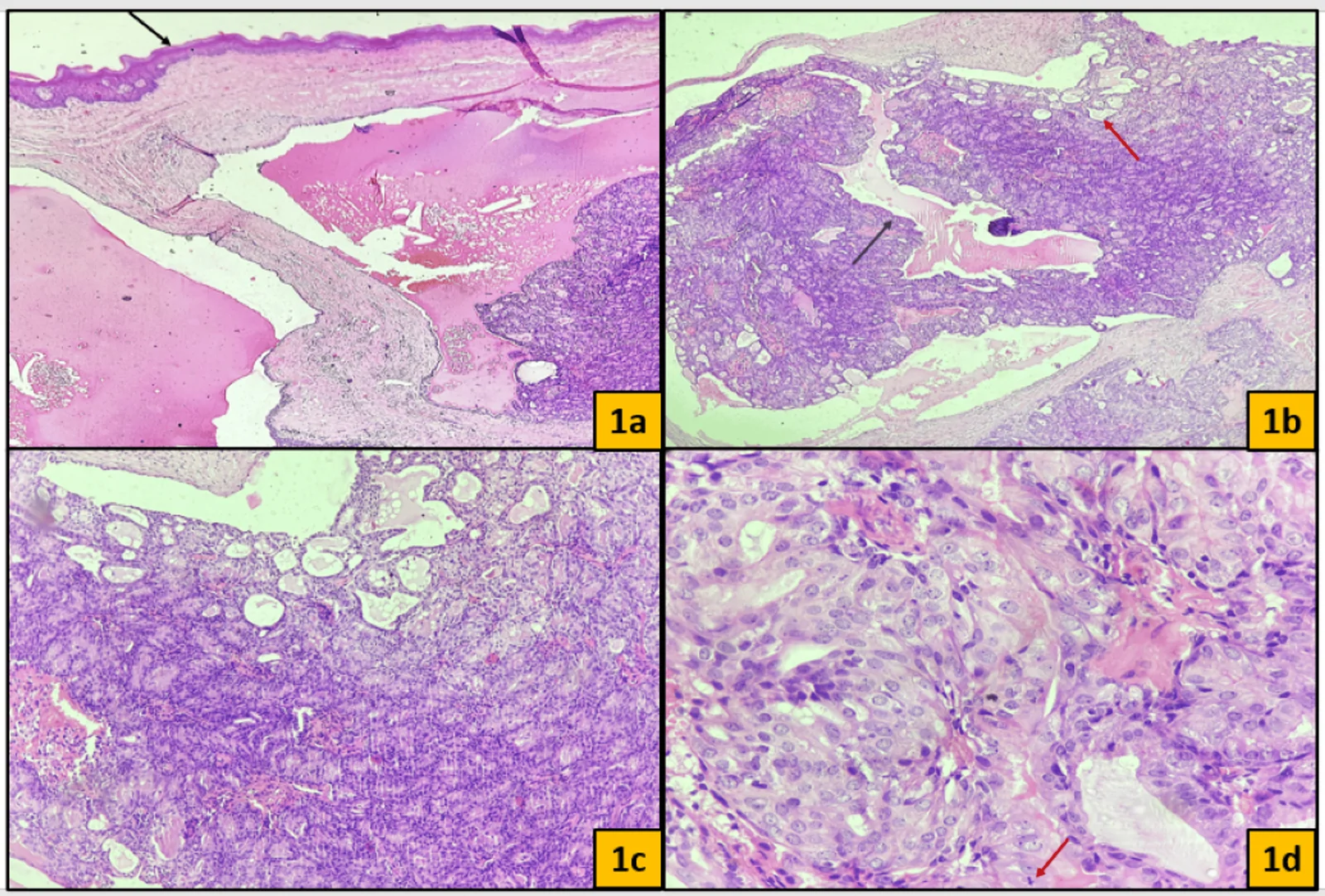
Histopathology findings. (a, b) Hematoxylin and eosin ×40: Tissue lined by stratified squamous epithelium (black arrow) with subepithelial tissue shows the presence of tumor in a macro (blue arrow) and microcystic pattern (red arrow) and lobules with eosinophilic secretions in the lumen. (c, d) The individual tumor cells showed prominent acinar pattern with moderate amount of cytoplasm and rare mitosis (red arrow).

Based on histological features, the following differential diagnoses were considered: SC, acinic cell carcinoma (AcCC), low-grade intraductal carcinoma, low-grade mucoepidermoid carcinoma, and polymorphous adenocarcinoma, with SC and AcCC being the most common. On immunohistochemistry (IHC), the tumor cells were positive for Mammaglobin, GATA3, S100, and CK7 and negative for DOG1 (Figure 2).

**Figure 2:**
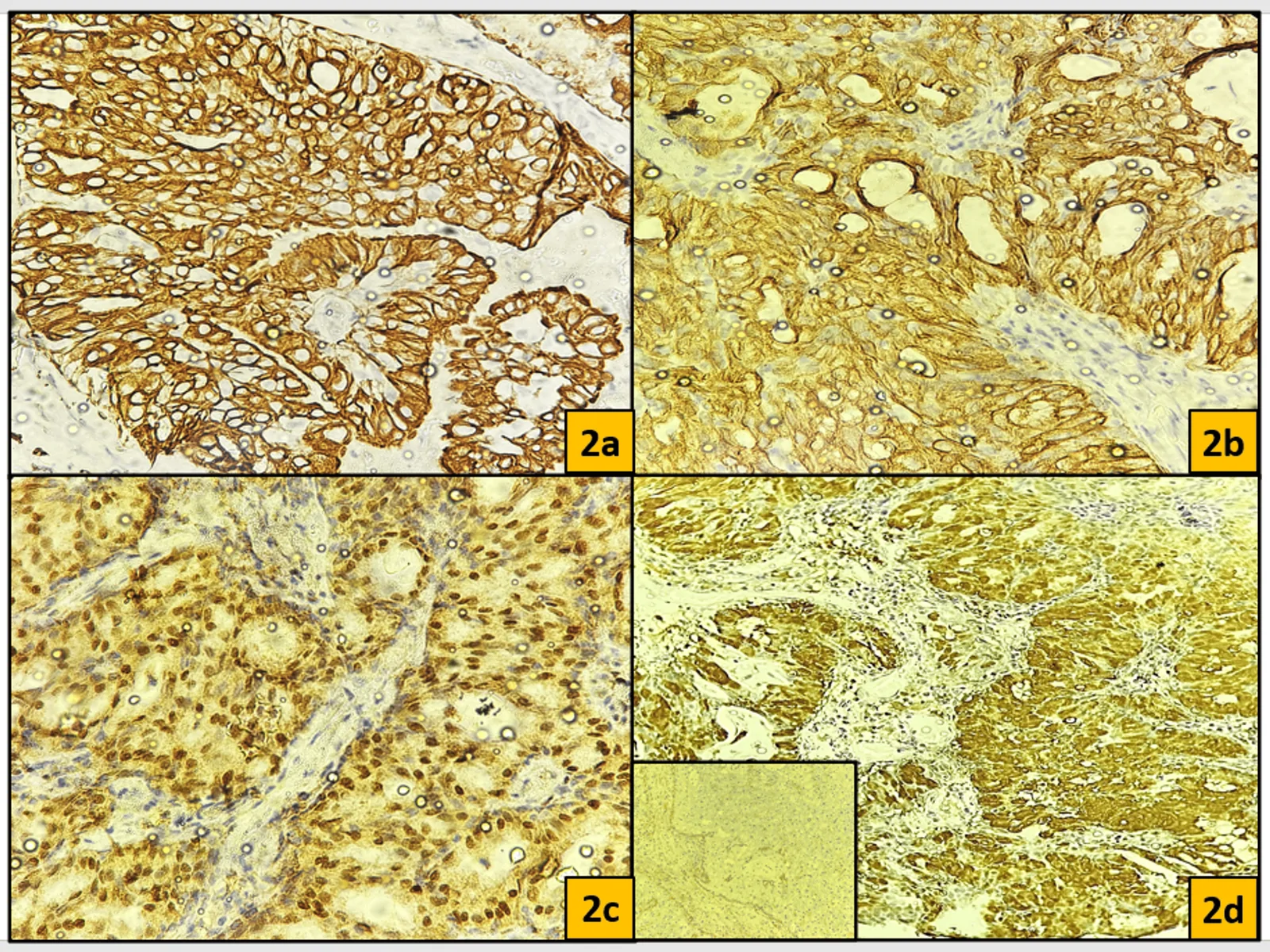
Immunohistochemistry findings. Immunohistochemistry ×200: The tumor cells are positive for mammaglobin (a), CK7 (b), GATA3 (c), and S100 (d), with inset showing negative DOG1.

Based on histomorphological and IHC findings, a diagnosis of SC was made. Molecular testing for *ETV6-NTRK3* fusion transcripts could not be performed due to unavailability. Postoperative positron emission tomography-computed tomography revealed no tumor deposit. The patient had remained recurrence-free until the last follow-up at six months post-surgery.

## Discussion

Intraoral salivary gland malignancies account for approximately 6% of all head and neck malignancies, whereas SC represents fewer than 0.3% of salivary gland malignancies [4]. SC is an uncommon low-grade malignant epithelial tumor that closely resembles SC of the breast in both its histopathological appearance and molecular profile. Although first described as a separate salivary gland neoplasm in 2010, it was formally incorporated into the WHO classification of head and neck tumors as a distinct entity in 2017. It predominantly affects middle-aged adults, with a reported mean age at diagnosis of approximately 45 years [2,5]. The parotid gland is the most frequent site of involvement, followed by the oral cavity and the submandibular gland. Among intraoral locations, the lip and soft palate are the sites most commonly affected [3]. Previously reported cases involving the buccal mucosa are summarized in Table 1 [1,6-25].

**Table 1:** Cases of secretory carcinoma of the buccal mucosa in the literature. SC: secretory carcinoma; BM: buccal mucosa; M: male; F: female; LVI: lymphovascular invasion; PNI: perineural invasion; HGT: high-grade transformation; LN: lymph node; N/A: not available

Study	Year	Age/Gender	Number of SC in the BM/Total number of cases of SC in the study	LVI/PNI/Metastasis	HGT
Skálová et al. [6]	2010	51/F	1/16	Absent	Absent
Fehr et al. [7]	2011	43/M	1/4	--	--
Chiosea et al. [8]	2012	--	2/36	Absent	Absent
Bishop et al. [9]	2013	71/M	1/11	Absent	Absent
Keisling et al. [10]	2014	5/F	1/1	Absent: loss to follow-up	Absent
Serrano-Arévalo et al. [11]	2014	41/F and 50/M	2/4	N/A	N/A
Din et al. [12]	2016	--	1/11	Absent, recurrence present	Absent
Khurram et al. [13]	2016	--	1/11	N/A	N/A
Skálová et al. [14]	2016	31/F and 24/F	2/25	--	--
Kai et al. [15]	2017	58/M	1/1	Absent	Absent
Guilmette et al. [16]	2017	64/F	1/7	LVI present/PNI absent	Absent
Zhao et al. [17]	2018	--	1/6	N/A	N/A
Paudel et al. [18]	2019	54/F	1/1	Absent	Absent
Hedhli et al. [19]	2020	--	1/1	N/A	N/A
Alramadhan et al. [20]	2020	47/M	1/1 (1/66)	N/A	N/A
Ogawa et al. [21]	2021	84/M	1/2	Absent	Absent
Asai et al. [22]	2021	44/M	1/1	Metastasis present: lung	Present
Xu et al. [1]	2022	--	7/90	LVI present (1/7, 33/F), PNI present (1/7, 44/M)	Present (1/7)
Langah et al. [4]	2023	42/M	1/1	Level I cervical LN metastasis	Absent
Durzyńska et al. [23]	2023	59/F	1/6	Absent	Absent
Chida et al. [24]	2024	46/M	1/1	Absent	Absent
Mahomed et al. [25]	2024	40/M and 70/F	2/7	Nodal metastasis, PNI present, LVI present in the first case and absent in the second case	Absent

SC of the buccal mucosa was identified in approximately 32 patients in the published literature. The mean age was 48.6 years, similar to SC of other sites, with no sex predilection. Although SC is a known low-grade malignancy, certain clinicopathological factors associated with poor prognosis have been noted in cases reported in the buccal mucosa. LVI was noted in 9.3% of cases (3/32 cases), whereas PNI was noted in 6.2% of cases (2/32 cases). Metastasis was identified in 9.3% of cases (3/32 cases), of which two cases showed nodal metastasis and one case showed metastasis to the lung. High-grade transformation, characterized by solid growth, nuclear anaplasia/pleomorphism, and prominent geographic necrosis, was noted in 6.2% of cases (2/32 cases) (Table 1). Cases with high-grade transformation are frequently associated with an increased risk of metastasis [1].

The differential diagnosis of SC includes AcCC, low-grade intraductal carcinoma, low-grade mucoepidermoid carcinoma, and polymorphous adenocarcinoma, with AcCC being the most common because of histopathological similarities (Table 2) [2].

**Table 2:** Differential diagnoses of secretory carcinoma.

Diagnosis	Histomorphological features	Immunohistochemistry/Molecular abnormality
Acinic cell carcinoma	Solid, microcystic, follicular, and papillary cystic architectures with a prominent lymphoid stroma. Serous acinar cells with PAS+ basophilic zymogen granules, with variable intercalated duct-type, vacuolated, oncocytic, or rarely clear cells	SOX10 and DOG1+, NR4A gene rearrangements
Low-grade (intercalated and oncocytic) intraductal carcinoma	Intercalated IDC: Amphophilic to eosinophilic cells with oval nuclei. Oncocytic IDC: Abundant, granular eosinophilic cytoplasm and round nuclei with prominent nucleoli	S100, mammaglobin and SOX10+, *NCOA4-RET* fusion genes (intercalated) and *TRIM33-RET* fusion (oncocytic)
Low-grade mucoepidermoid carcinoma	Circumscribed, partly cystic, and contains groups of mucous cells	P63 and p40+, *CRTC1-MAML2* fusion gene
Polymorphous adenocarcinoma	Wide variation of architectural patterns; the tumor cells are uniform in shape, with scant cytoplasm; and bland oval nuclei with nuclear grooves. Streaming or eddy-like formations can be seen at the periphery of the tumor. Perineural involvement frequent	CK7, S100, p63, mammaglobin, CD117, CEA+, *PRKD* gene alterations
Secretory carcinoma	Well-circumscribed, lobulated growth pattern separated by fibrous septa, and composed of solid, microcystic, follicular, and papillary cystic architectures with distinct luminal secretions. The cells have low-grade vesicular round-oval nuclei and centrally located nucleoli. Cytoplasm is granular to vacuolated. Mitoses are rare	CK7, S100, SOX10, vimentin, mammaglobin+, *ETV6*-*NTRK3* fusion gene

The presence of papillary, cystic, and microcystic patterns with vacuolated cells is usually characteristic of SC. Hemosiderin deposition is also more commonly seen in SC than in AcCC [14].

SCs are characterized by recurrent *ETV6* or *RET* gene rearrangements, with *ETV6::NTRK3* and *ETV6::RET* fusion transcripts identified in the majority of tumors [3]. Current evidence does not establish *ETV6::NTRK3* fusion status itself as an independent prognostic factor in secretory carcinoma. Other parameters, including high mitotic activity, necrosis, high-grade transformation, LVI, positive margins, and advanced stage, appear more relevant to prognosis [1]. Although molecular confirmation would have provided additional diagnostic certainty and potentially established eligibility for *NTRK*-targeted therapy in the present case, its absence is unlikely to significantly alter management in a localized, completely excised tumor.

Immunohistochemically, SC typically demonstrates diffuse and strong mammaglobin positivity, making it a valuable diagnostic marker. Mammaglobin belongs to a family of secretory glycoproteins that are physiologically expressed in normal mammary epithelium and are frequently overexpressed in breast carcinoma. Its expression in salivary gland tumors suggests the presence of a mammary analogue secretory component in the tumor cells. In contrast, DOG1 is predominantly localized to normal salivary gland tissue, particularly along the apical membrane of acinar cells. Therefore, the absence of DOG1 immunoreactivity, as observed in the present case, favors a diagnosis of SC and helps exclude AcCC [14].

## Conclusions

SC is a rare salivary gland neoplasm, occurring predominantly in the major salivary glands and less frequently in the minor salivary glands of the oral cavity. Involvement of the buccal mucosa is particularly uncommon. Accurate diagnosis can be achieved in most cases through careful histomorphological evaluation supplemented by an appropriate IHC panel. Molecular genetic testing for characteristic gene fusions may be reserved for diagnostically challenging or atypical cases.
